# Biogeographic distributions of neotropical trees reflect their directly measured drought tolerances

**DOI:** 10.1038/s41598-017-08105-8

**Published:** 2017-08-21

**Authors:** Adriane Esquivel-Muelbert, David Galbraith, Kyle G. Dexter, Timothy R. Baker, Simon L. Lewis, Patrick Meir, Lucy Rowland, Antonio Carlos Lola da Costa, Daniel Nepstad, Oliver L. Phillips

**Affiliations:** 1 School of Geography, University of Leeds, Leeds, LS2 9JT UK; 2 Royal Botanic Garden of Edinburgh, EH3 5LR Edinburgh, UK; 3 School of Geosciences, University of Edinburgh, Edinburgh, UK; 4 Department of Geography, University College London, London, UK; 5 Research School of Biology, Australian National University, Canberra, Australia; 6 College of Life and Environmental Sciences, University of Exeter, Exeter, UK; 7 Universidade Federal do Pará, Belém, Pará, Brazil; 8 Earth Innovation Institute, San Francisco, CA USA

## Abstract

High levels of species diversity hamper current understanding of how tropical forests may respond to environmental change. In the tropics, water availability is a leading driver of the diversity and distribution of tree species, suggesting that many tropical taxa may be physiologically incapable of tolerating dry conditions, and that their distributions along moisture gradients can be used to predict their drought tolerance. While this hypothesis has been explored at local and regional scales, large continental-scale tests are lacking. We investigate whether the relationship between drought-induced mortality and distributions holds continentally by relating experimental and observational data of drought-induced mortality across the Neotropics to the large-scale bioclimatic distributions of 115 tree genera. Across the different experiments, genera affiliated to wetter climatic regimes show higher drought-induced mortality than dry-affiliated ones, even after controlling for phylogenetic relationships. This pattern is stronger for adult trees than for saplings or seedlings, suggesting that the environmental filters exerted by drought impact adult tree survival most strongly. Overall, our analysis of experimental, observational, and bioclimatic data across neotropical forests suggests that increasing moisture-stress is indeed likely to drive significant changes in floristic composition.

## Introduction

The future composition and structure of tropical forests may be substantially altered by periods of high moisture stress, such as droughts. Well-known macroecological diversity patterns of tropical trees and lianas strongly suggest that water supply, particularly the length and severity of the dry season, is a major constraint on plant survivorship: woody plant diversity is greatest where seasonal moisture deficits are lowest^1, 2^. This implies that many tropical woody taxa are ultimately limited by physiological constraints related to water supply, and that the distribution of individual taxa over biogeographical gradients of moisture availability could predict their vulnerability to droughts^3, 4^. Indeed, the current climatic distributions of taxa are commonly presumed to predict species’ responses to future climate change in the tropics and beyond e.g. refs 5, 6–8, - and yet we do not know whether these distributions reflect actual climatic tolerances. Given the exceptional diversity of tropical forests and the likelihood of increased moisture stress^9–11^, it is vitally important to test empirically whether large-scale floristic distributions do indeed provide information on the tolerance of different taxa to extreme climatic conditions.

The hypothesis that physiological tolerance to moisture stress drives the distributions of tropical tree species is supported by evidence from experimental work where seedlings of several taxa were exposed to drought conditions^4, 12, 13^. However, these seedling experiments may not necessarily represent the overall drought-vulnerability of tropical trees. For instance, the very controlled conditions in which seedlings are grown experimentally do not include interactions among species, such as above- and belowground competition. The drought tolerance of seedlings is also expected to differ from that of adult trees: whilst seedlings have limited water and carbon storage, potentially making them more vulnerable to drought than trees^14^, they are protected by several canopy layers and thus less exposed to high radiation loads and temperatures. Additionally, fundamental functional differences among species that translate into variations in drought vulnerability may not be expressed in seedlings. Recent research has, for example, demonstrated that drought resistance is often related to tree size, with taller trees being more vulnerable to drought-induced mortality^15–19^. Taller trees are more likely to have more vulnerable tissues, which increases the chances of hydraulic failure^19^ leading to tissue desiccation and potentially death^14^.

As an alternative to the experimental approach, evaluating the impacts of actual tropical droughts on forests may provide broader insights into hydrological limitation in tropical trees, especially because the sample size constraints faced by seedling experiments may be reduced. For example, temporal trends in community composition at regional and local scales among permanent sample plots have shown that droughts in the tropics favour species affiliated to drier local environments^20–23^. For instance, species that occur under the driest conditions along a precipitation gradient (1800–4000 mm y^−1^) across the Isthmus of Panama have undergone a relative increase in abundance in Barro Colorado Island, after a long-term increase in aridity^21^. Similar results were observed in Ghana at the lower end of the rainfall gradient (1000–1800 mm y^−1^) in a study of 21 plots distributed across that country’s forest zone, which represents the largest scale study of this question in the tropics^22^. Although these studies suggest that the distribution vs. tolerance relationship holds for tropical forests at local scales, no direct analysis of this relationship that covers a broad range of precipitation values and different life history stages has yet been attempted. Such an approach would provide insights into the potential responses of tropical forest taxa to increasing moisture stress.

Large parts of the neotropical forest realm have recently experienced increased moisture stress, driven by a decrease in precipitation in some regions, widespread extreme drought events and a general rise in temperature^24–26^. Total precipitation has declined recently in Central America and in the southern borders of the Amazon basin e.g. refs 21, 27. At the same time, the frequency of extreme dry events has increased, with the 2005, 2010, and 2015 droughts affecting much of the Amazon^28–31^. The dry season has also recently lengthened along the southern border of Amazonia^26^, and this trend is likely to continue as an outcome of both deforestation^32^ and global climatic changes^10, 11, 33^. Even in neotropical regions where precipitation has increased, notably the northwest of the Amazon basin^9, 34^, plants may also experience higher moisture stress, now and in the future, as a consequence of actual and projected increases in temperature and precipitation seasonality^24^. Increasing moisture stress may be partially responsible for the observed long-term increase in mortality rates in Amazonian trees^35^, and is clearly linked to mortality spikes during and after droughts^17, 36–38^. Given the predictions of drier conditions, better delimitation of the drought vulnerability of different tree taxa would improve our understanding of how tropical forest communities will respond to future changes in climate.

**Figure 1:**
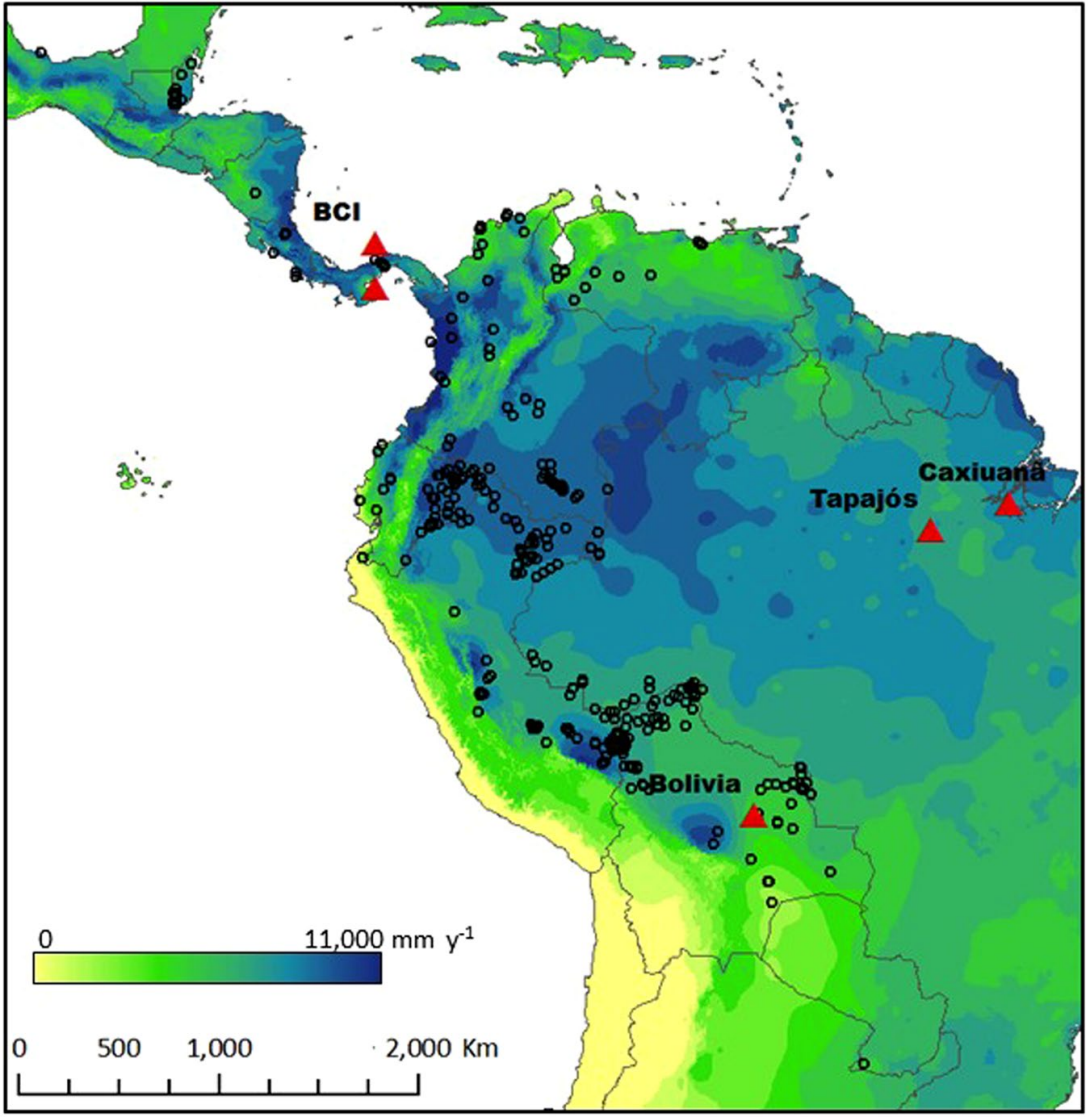
Location of experiments and observations analysed in this study. Triangles represent case studies investigated: two through-fall exclusion experiments in the Brazilian Amazon, Tapajós^40^ and Caxiuanã^16^, field observations from the 1982–83 El Niño drought^17^, a seedling drought experiments in Panama (BCI)^4^ and  a seedling drought experiments in Bolivia^12^. Circles show ForestPlots.net^66, 78^ and ATDN^1^ inventory plots used to calculate water deficit affiliation (WDA) in ref. 39. Patterns within the map represent mean annual precipitation in mm y^−1^ from WorldClim^79^ generated in ref. 80. Note that case studies are located across a range of precipitation regimes and that Tapajós and Caxiuanã are ≈2000 km away from the area where WDA was calculated, allowing us to explore whether geographically distant populations will respond in the same way to droughts.

Here we investigate the relationship between the large-scale bioclimatic distribution of genera and their tolerance to droughts at different life-history stages. We use information on water deficit affiliation (WDA) from ref. 39, which quantifies the affiliations of genera to different precipitation conditions across a wide precipitation gradient (500–3500 mm y^−1^). This variable represents the ‘centre of gravity’ of species abundance across a gradient of water deficit by assessing relative abundances in 531 floristic plots of ~1 ha each distributed across the western Neotropics, thus weighting towards the precipitation conditions where each taxon shows its greatest relative abundance (Fig. 1). To quantify drought tolerance, we assessed five drought events, both experimental and natural, spanning different life-history stages and distinct regions within the Neotropics (Table 1). These include two through-fall exclusion (TFE) experiments conducted in the Brazilian Amazon, at (1) Tapajós^40^ and (2) Caxiuanã^41^, (3) one observational study conducted over the 1982 drought in a 50 ha tree inventory plot on Barro Colorado Island in Panama^17^, hereafter referred to as BCI, and the results from two shade house experiments testing drought sensitivity on transplanted seedlings, in (4) Panama^4^ and (5) Bolivia^12^.

**Table 1:** Description of through-fall exclusion experiments and field observations analysed in this study.

Experiment	Life stage	Mean annual rainfall (mm y^−1^)	Cumulative water deficit (mm y^−1^)	% rainfall exclusion	Duration (y)	Area (ha)	N_gen_	n_0_	D_dry_
**Natural drought**									
(1) 1982 BCI El Niño drought^17^	trees	2493	−311	59	0.3	50	99	20282	1772 (9%)
	saplings						141	213106	17671 (8%)
**Through-fall exclusion experiments**									
(2) Tapajós^40^	trees	2024	−324	50	4.2	1	82	311	59 (19%)
	saplings						123	2116	179 (8%)
(3) Caxiuanã^16^	trees	2187	−225	50	7	1	74	314	50 (16%)
**Seedling experiments**									
(4) Panama^4^	seedlings	2493	−311	100	0.4	NA	45	1411	482 (42%)
(5) Bolivia^12^	seedlings	1023	−699	100	0.5	NA	31	1440	1440 (100%)

N_gen_ represents the number of genera examined in each drought experiment; n_0_ is the number of individuals before the drought in the droughted area. D_dry_ shows the number of deaths during the drought. Life history stage represented by trees (≥100 mm diameter), saplings (10–99 mm D in BCI and 20–99 mm in Tapajós), and seedlings.

By their nature, manipulative experiments in the diverse neotropical forest environment, even when at the “ecosystem” scale, are typically able to only sample a few individuals of any given taxon. For this reason, our ability to infer drought sensitivity of any one taxon from any one experiment is necessarily limited. Furthermore, each experiment has a unique set of environmental and biological conditions. Therefore, our approach was to examine responses, to the extent possible, across all genera, combining available experimental and observational data on drought-induced mortality. We hypothesize that biogeographically wet-affiliated neotropical tree genera will prove to be more sensitive to drought conditions. We predict that drought-related traits should be more important for trees in their adult phase; thus, dry-affiliated genera should have a greater advantage as adults than as seedling and saplings. Additionally, we predict that the longer the drought in a given study, the stronger the relationship between WDA and drought-induced mortality, as longer droughts lead to higher tree mortality, especially of drought-vulnerable genera^16, 42^.

## Results

Overall, the water deficit affiliations (WDA) of neotropical tree genera, which display more strongly negative values for genera that occur in drier conditions, are significantly associated with their tolerances to drought.

**Figure 2:**
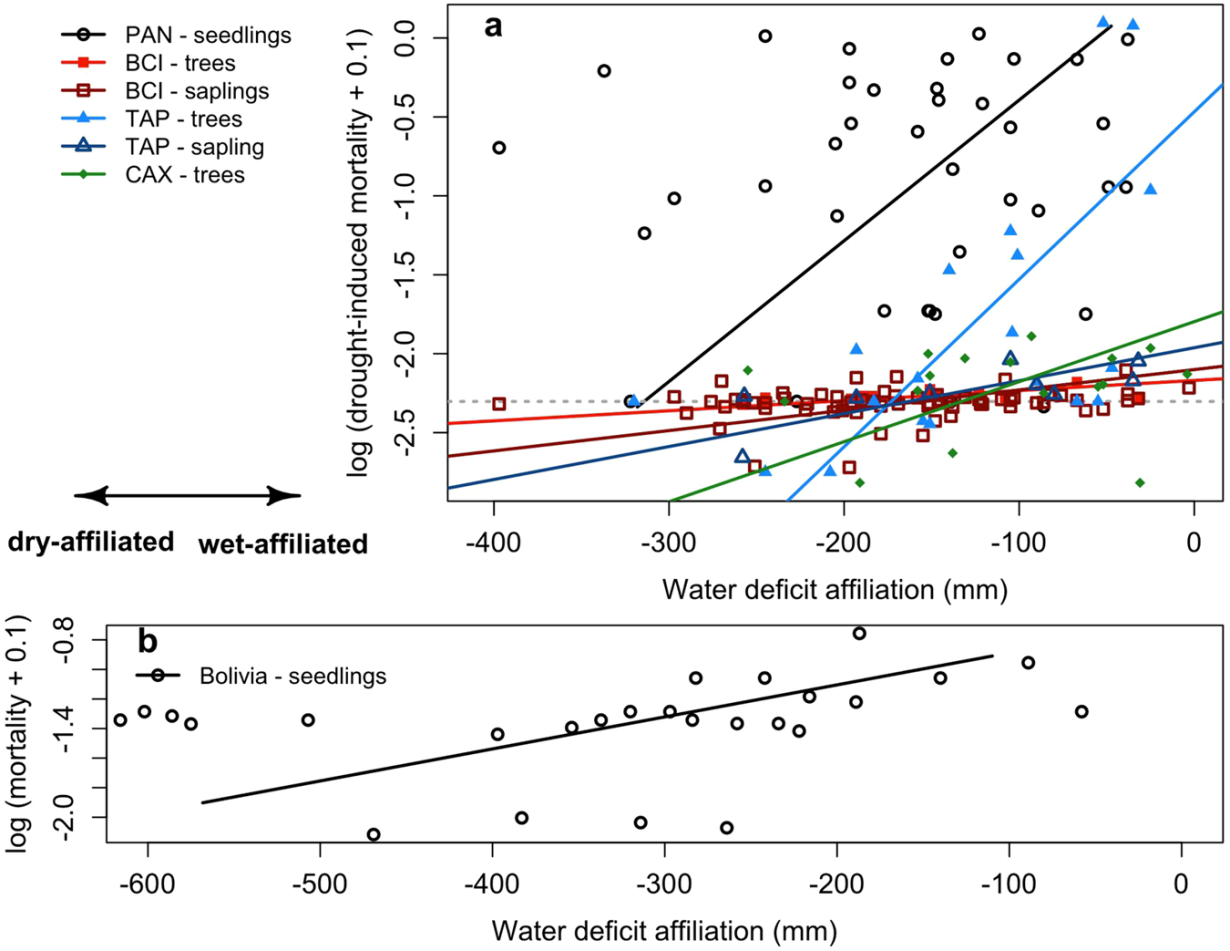
Standardized major axis regression (SMA) between log-transformed drought-induced mortality (∆m) and water deficit affiliation (WDA) in different case studies and for different life history stages. (**a**) Two through-fall exclusion experiments in the Brazilian Amazon, Tapajós^40^ and Caxiuanã^16^, field observations from the 1982–83 El Niño drought^17^ and a seedling drought experiment in Panama^4^; grey horizontal line indicates zero ∆m, so that genera above this line experienced greater drought mortality than baseline mortality. (**b**) Seedling drought experiment in Bolivia^12^, where y-axis represents log transformed mortality rates (m) +0.1 during the drought experiment. Trees include stems over 100 mm diameter. The size range of saplings sampled was 10–99 mm D in BCI and 20–99 mm in Tapajós. WDA data were extracted from ref. 39.

In each of the case studies, the WDA of genera was positively correlated with drought-induced mortality when assessed using Kendal’s τ, and was significantly or marginally significantly in all cases, except for trees in Caxiuanã and the seedling experiment in Panama (Table 2). The slopes of a standardized major axis regression are also positive in all cases, and significant for Tapajós (trees and saplings) and marginally significant for the seedling experiment in Panama and the natural drought in BCI (Fig. 2a; Table 2).

**Table 2:** Relationship between drought mortality (∆m) and water deficit affiliation (WDA) in five droughted locations in the Neotropics.

Source	Life stage	slope	intercept	Kτ	Number of genera	Min. number of stems per genus
BCI	Trees	6 × 10^−4+^	2.2	0.45*	12	730
	Saplings	1 × 10^−3+^	−2.1	0.14^+^	69	602
Tapajós	Trees	0.01**	−0.5	0.44**	19	8
	Saplings	2 × 10^−3^*	−2	0.61*	9	100
Caxiuanã	Trees	4 × 10^−3^	−1.8	0.16	17	6
Panama	Seedlings	9 × 10^−3+^	0.5	0.05	40	53
Bolivia	Seedlings	2 × 10^−3^	−0.7	0.32*	31	40

Slope and intercept from standardized major axis regressions (SMA) and Kendall’s τ coefficient of correlation (Kτ) between ∆m and WDA were calculated for the 1982–3 El Niño drought in BCI^17^, two throughfall exclusion experiments in the Brazilian Amazon, Tapajós^40^ and Caxiuanã^16^, and two seedling experiments, one in Panama^4^ the other in Bolivia^12^. For BCI, Tapajós and Caxiuanã the relationship ∆m *vs*. WDA was calculated for the subset (number of genera), including only genera with the minimum number of stems needed to permit estimation of mortality for that experiment (see Supplementary methods S2). The minimum number of stems per genus varied depending on the duration of the drought (Supplementary methods S2). One-tail P-values test (1) whether the slope differs from zero and (2) the null hypothesis of a positive relationship (positive values of Kτ) between ∆m *vs*. WDA. The latter analysis was repeated using two-tailed tests, which showed no difference on the results (Supplementary Table S6). ^+^P < 0.1, *P < 0.05; **P < 0.01.

The relationships between WDA and drought-induced mortality were maintained even when accounting for the influence of phylogenetic autocorrelation. When the mortality data from all experiments is combined into one statistical model controlling for the effects of phylogeny, the coefficient associated with effect of WDA on mortality (∆m) is positive, with credible intervals that do not overlap zero (Table 3).

**Table 3:** Relationship between drought-induced mortality (∆m) and water deficit affiliation (WDA) for Neotropical tree genera after accounting for experiment and phylogenetic autocorrelation using Bayesian phylogenetic mixed models.

Model	slope	lower CI	upper CI
intercept	0.6	−4 × 10^−2^	1.1
WDA	3.5 × 10^−3^	4.5 × 10^−4^	6.3 × 10^−3^
experiment*			
Caxiuanã	−0.3	−1.3	0.6
Panama	−0.4	−1.3	0.4
Tapajós	0.3	−0.5	1.2
**WDA: experiment**			
Caxiuanã	−1.2 × 10^−3^	−8.5 × 10^−3^	5.6 × 10^−3^
Panama	−2.5 × 10^−3^	−6.8 × 10^−3^	1.8 × 10^−3^
Tapajós	3.4 × 10^−3^	−2 × 10^−3^	8.5 × 10^−3^

The model was fitted for all studies that had a control treatment (i.e. all except Bolivia). Values of ∆m were standardized as z-scores for each experiment. Lower and upper CI represent respectively the lower and upper 95% credible intervals for the slope parameter. The model used an uninformative inverted gamma distribution prior for each parameter, following ref. 75. Posterior distributions were obtained from 260,000 iterations, a burn-in = 60,000, and thinning interval = 200 ref. 75. Note that WDA shows a positive relationship with ∆m with credible intervals never overlapping zero. *Case studies were dummy coded; BCI was coded zero for each variable.

Life history stage also influenced drought-induced mortality of tree genera. Firstly, those models including the interaction between life history stage and WDA showed lower deviance information criteria (DIC) values than simpler models when using the data of BCI and Tapajós, the two sites where both trees and saplings were sampled (Table 4). Secondly, for the best model in Table 4, life stage and its interaction with WDA significantly explained drought-induced mortality (posterior mean = 0.003; 95% CI = 0.0006, 0.005). In addition, in Tapajós, the only throughfall exclusion experiment in which both saplings and trees were studied, the ratio of drought:control mortality was four times greater for trees than saplings (see Supplementary Table S4).

**Table 4:** Comparison of Bayesian phylogenetic mixed models describing the relationship between drought-induced mortality (∆m) and water deficit affiliation (WDA) for Neotropical tree genera.

Fixed effects	DIC	∆ DIC
WDA + experiment + life stage + WDA:experiment + WDA:life stage	57	0
WDA + experiment + life stage + WDA:experiment	62	5
WDA + experiment + life stage + WDA:life stage	62	5
WDA + experiment + life stage	83	26
WDA + experiment	86	29
WDA + life stage	87	30
experiment + life stage	88	31
experiment	92	36
life stage	93	36
WDA	99	42
Null model	110	53

Models vary in how they account for the influence of experiment (Tapajós or Barro Colorado Island) and life-history stage (saplings or trees) and are compared based on deviance information criteria (DIC). Data on ∆m were obtained from the Tapajós through-fall exclusion experiment^40^ and from the 1982 El Niño drought in BCI^17^. WDA values were taken from ref. 39. Models based on an uninformative prior inverted gamma distribution, following ref. 75. Posterior was obtained after 130 000 iterations, burn-in = 30 000 and thinning interval = 100 ref. 75. Note that the best models are the ones including WDA, experiment and life history stage, and the interactions of WDA with experiment and life stage.

Our results allowed us to identify the vulnerability of particular taxa. Drought-mortality was greater than baseline mortality across more than one experiment for 22 of the 51 genera that occur in at least two different studies (Supplementary Table S5). Among these vulnerable genera, we highlight *Inga* and *Hymenaea* (Leguminosae), *Pouteria* (Sapotaceae), *Casearia* (Salicaceae), *Guarea* (Meliaceae), *Virola* (Myristicaceae), *Licaria* (Lauraceae) and *Eschweilera* (Lecythidaceae). Only five genera (*Capparidastrum* [Capparaceae], *Faramea* [Rubiaceae],  *Garcinia* [Clusiaceae], *Socratea* [Arecaceae] and *Miconia* [Melastomataceae]) were consistently resistant, with drought treatment mortality rates lower than or equal to the baseline mortality.

## Discussion

We compared the bioclimatic distribution of 115 tree genera along a continental scale moisture gradient with drought-induced mortality rates from experimental and natural drought events across the Neotropics. There is a positive relationship between the degree of affiliation to high climatic water supply (WDA) of genera and the degree to which droughts increase their mortality rates. Drought selectively kills genera that are predominantly found in wetter climates (Fig. 2; Table 2), even after accounting for phylogenetic autocorrelation (Table 3). These findings represent the first demonstration at continental scales of relationships so far inferred from seedling studies at local to regional scales^4, 12, 22, 43^. Furthermore, the relationship between drought-induced mortality and biogeographic WDA was stronger for trees when compared to saplings, providing evidence that selective drought filters may be stronger for larger size classes.

These results demonstrate that the climatic distribution of tropical tree genera can explain their actual, measured demographic vulnerability to moisture stress. Of the five experimental and observational drought mortality studies examined, two were located at least 2000 km distant from where water deficit affiliations were measured. This suggests that genera maintain a similar level of adaptation to drought wherever they occur in moist forests in the Neotropics. The fact that the bioclimatic distribution of tree genera across moist forests in the Neotropics is related to their tolerance to droughts also suggests that, to some degree, distributions reflect fundamental niches. Therefore, our results corroborate the central idea behind species distribution modelling, for which current taxonomic distribution is assumed to provide proxy information on physiological tolerances to climatic extremes e.g. refs 44, 45.

The predictive power of these relationships should however be interpreted with caution. The strong correlations of taxonomic diversity with rainfall^2^ and the distribution of genera across precipitation regimes^39^ argue for the primacy of moisture effects at the large, continental spatial scales considered here. Nevertheless, biogeographic distributions are shaped by a combination of factors. In the lowland tropics, as well as drought-tolerance, biological interactions^46, 47^, edaphic conditions^48–50^, shade tolerance^12^ and dispersal^51–54^ may all influence biogeographic distributions. Moreover, the scarce  available information on taxon-specific tree mortality, and the exceptional high species richness in the tropics, add complexity when testing for relationships between drought-induced mortality and biogeographical distributions. The high diversity of these forests hampers us from performing analyses at the species-level, where we might expect a stronger association between taxon and climate. This is suggested in the contrast between our results for the seedling experiment in Panama, where a weak relationship between drought-induced  genus-level mortality and WDA was found, and the findings from ref. 4, where regional species-level distributions did significantly explain drought-mortality. Additionally, although the relationships shown are consistent across case studies and with our predictions, they are based on the relatively few genera, those for which drought-induced mortality estimates were available (Table 2). We further note that drought-experiments in the Neotropics are located in seasonal areas (Table 1), which potentially restricts our understanding of drought-tolerance to more drought-resistant forests and undersamples those genera which are affiliated to aseasonal conditions^39^ (Supplementary Fig. S7). The lack of drought experimentation in the least seasonal neotropical forests needs to be addressed to deepen our understanding of potential drought impacts on the neotropical flora.

Drought acts more strongly as an environmental filter for adult trees. Our analyses showed that dry-affiliated genera are disproportionately favoured in dry environments and that this advantage is greater among adult trees than seedlings or saplings. Tree size is an important predictor of drought vulnerability^15–19, 40^. Recent work at the Caxiuanã throughfall exclusion experiment has identified hydraulic failure as the most likely trigger of drought-induced mortality in large trees^19^. All things being equal, the larger the tree is, the greater the risk is of hydraulic failure, as the vulnerability of xylem tissue to cavitation increases with tree size^19^, and tall crowns are more exposed to high temperature and radiation, substantially increasing evaporative demand^42, 55^. Consequently, our findings suggest that traits related to resistance to hydraulic failure - such as wood density, vessel size and vessel density^56, 57^ and the capacity to close stomata during dry periods^16, 42, 55, 58^ - may provide a greater relative advantage in adult trees exposed to high vapour pressure deficits. Clearly such an advantage would be less important for seedlings or saplings.

Overall, the impact of drought on mortality increases with the duration of the drought. Thus, the drought:control mortality ratio increased from BCI to Tapajós to Caxiuanã, which experienced increasingly longer droughts (see Supplementary Table S4). Nevertheless, for the longest drought (7 years of reduced through-fall in Caxiuanã), climate affiliation loses its power to predict mortality amongst genera, with a steeper slope to the relationship between drought-induced mortality and WDA for the shorter Tapajós experiment (Table 2). It appears that over extended periods, drought-resistant genera also eventually experience significant mortality, translating to a less strong relationship between ∆m and WDA (Table 2; Fig. 2). If so, this would indicate that after 7 years of artificial drought most genera have exceeded their moisture deficit tolerance, at which stage basic ecosystem services such as biomass carbon storage begin to collapse^59^. In this scenario, most trees would eventually have died, regardless of their WDA tolerance. The implication is that while forest biodiversity, i.e. the functional and taxonomic diversity found in a community, may provide ecosystem-level resilience to short-term droughts, there exist thresholds of drought intensity and duration beyond which most or all tree genera suffer, heavily compromising ecosystem function.

Our analysis suggests that in the lowland Neotropics, droughts are likely to disproportionately impact biogeographically wet-affiliated tree genera. While the strength of this pattern varies based on drought duration and life history stage, it is consistent across studies, indicating the potential for severe natural droughts - such as those that occurred in 2005, 2010 and 2015 in Amazonia^29, 31, 60^ - to at least temporarily affect community composition through selective mortality. Our results suggest that a reassembly of Amazonian tree communities is likely to take place under drier climate conditions, just as has occurred recently in parts of West Africa^23^ where dry-affiliated genera have increased in abundance. It is unknown how large and how reversible the impacts on biogeochemical ecosystem services of such an Amazonian reassembly will be^61, 62^, but regardless, the impacts on our planet’s biodiversity could be profound. In the Neotropics, wet-affiliated genera tend to have substantially smaller distributional ranges^39, 46^, and also represent the majority of the tree diversity in the Neotropics^1, 39^. Consequently, if droughts drive communities in favour of compositional changes towards dry-affiliated genera, as observed in these experiments, the climate changes anticipated in the coming century will alter neotropical forest composition and potentially endanger much of their exceptional plant and animal diversity.

## Methods

### Water deficit affiliation

To characterize the climatic conditions preferred by genera we made use of ‘water deficit affiliation’ (WDA) values that represent the precipitation conditions where the relative abundance of each genus is greatest. This metric is conceptually analogous to the elevation centre of gravity^21, 63^, and represents an abundance-weighted mean of climatic values across sites where the taxon occurs. WDA was calculated for individual taxa at different taxonomic levels (species, genus and family) using all stems greater than 100 mm in diameter at ~1.3 m above the ground from a network of 531 *terra-firme* inventory plots distributed throughout Western neotropical lowland closed canopy forests by ref. 39. Here we use WDA calculated using the mean cumulative water deficit (CWD) per year of each site as the input variable. CWD was extracted from ref. 64 and is an estimate in millimetres per year of the cumulative difference between precipitation (P) and potential evapotranspiration (E) over the consecutive months (n) within a year when evapotranspiration is higher than precipitation (Eq. 1):

1 $$CWD=\sum _{n=1}^{12}Min\,(0,\,{P}_{n}-{E}_{n})$$

where P_n_ and E_n_ are respectively the total precipitation and evapotranspiration for a specific month within a year. CWD was calculated using data from the Climate Research Unit dataset^65^ between 1960–1990^64^. The more negative the value of CWD, the larger the water deficit, thus strongly negative values of WDA indicate affiliations to seasonally dry conditions, whilst WDA values close or equal to zero represent affiliations to ever-wet conditions.

For some particular taxa, WDA is not *a priori* expected to accurately reflect a taxon’s true moisture affiliation, and these special cases could potentially confound our analysis. Firstly, heavily commercially logged taxa, notably *Swietenia macrophylla* (mahogany) and *Cedrela odorata* have been nearly exterminated in much of their range, so that current distribution and abundance are unlikely to represent realized precipitation niches. Secondly, and more significantly, for those taxa which are affiliated to locally enhanced water supply (‘LEWS’) - either through river flooding or with local water supply strongly determined by topography (permanent or seasonal swamps) - their large-scale biogeography may be largely decoupled from climate. To identify which neotropical species are strongly LEWS-affiliated, we computed an index based on the Neotropics-wide plot sampling available in ForestPlots.net^66^, which include 881 floristically-identified plots under forests with different levels of locally enhanced water supply. The abundance per hectare of all genera in each plot was calculated. Plots were classified as being either LEWS (i.e. swamp, floodplain or seasonal) or non-LEWS (i.e. *terra firme*) plots. For each taxon we calculated the ratio between its abundance in LEWS and non-LEWS plots, standardized by the number of plots and plot area. LEWS-affiliated genera, defined as those with such ratios of >1:1, comprised 11% of the 544 genera analysed by ref. 39. Our subsequent analyses were performed excluding LEWS-affiliated taxa as well as *S. macrophylla* and *C. odorata*.

### Moisture manipulation experiments and natural drought events

Information on drought sensitivity was accessed from five studies (Table 1; Fig. 1):

#### Natural drought

(1) Field records from the most detailed observation to date of local forest responses to a natural drought in the tropics: the measurements from a 50 ha plot at Barro Colorado Island, Panama (BCI) from the 1982–83 El Niño drought event. It has an extensive sample size per taxon, but is unreplicated spatially and lacks a pre-drought baseline^17^.

#### Through-fall exclusion (TFE) experiments

The only two TFEs in the Neotropics are from: (2) Tapajós^40^ and (3) Caxiuanã^16^ (Fig. 1). TFEs control for other possible factors affecting mortality by the use of non-droughted control plots, but they have limited sample size per taxon and no real replication. They also lack realistic atmospheric conditions of true drought, with humidity, air temperature and vapour pressure deficits mostly reflecting prevailing forest-wide conditions, while in terms of rainfall exclusion, they likely present an extreme, worst case scenario.

#### Seedling experiments

These tested the resistance of seedlings to drought in (4) Panama^4^ and (5) Bolivia^12^ and provide detailed information on individual mortality, but are restricted to a limited number of species sampled at each site.

The two through-fall exclusion experiments in the Brazilian Amazon (Tapajós and Caxiuanã) used a similar design but adopted different through-fall exclusion periods (Table 1). The intensity of rainfall exclusion is similar amongst these two experiments (*ca*. 50%) and that which occurred under natural drought at BCI (*ca*. 59%). In BCI and Tapajós, saplings were also measured (10–99 mm D in BCI and 20–99 mm D in Tapajós), and here were analysed separately from trees (>100 mm D).

Different experiments report drought sensitivity in different ways; for example, the Bolivia seedling experiments killed all seedlings and report days of survival whilst the TFEs report annualized mortality rates. To facilitate the comparison and interpretation of the results we therefore standardized the metric of drought sensitivity across all studies investigated here. For Tapajós, Caxiuanã, BCI, and the Panama seedling experiment, the available data include information on the number of individuals per species that were exposed to drought and non-drought treatments. This allowed us to apply the same mortality model to those studies and calculate mortality indices for each taxon in each experiment under experimental and control conditions. We applied the mortality equation used by ref. 16 and 40 (Eq. 2):

2 $$m=1-{({n}_{t}/{n}_{0})}^{1/t}$$

Eq. 2 estimates overall mortality rate of a population given the lapsed time in years, *t*, the number of stems at the end of the census interval, *n*_*t*_, and the number of stems in the first measurement, *n*_0_^67^.

In the original study that published the BCI data^17^, *t* was obtained from the average interval between censuses in each of different 20 × 20 m subplot within the 50 ha plot. However, information on the length of census intervals for different subplots is not available in ref. 17, we therefore used the overall average interval, 3.9 years for the drought period (1982–1985) and 5.25 years for the non-drought period (1985–1990). This approach generates almost identical mortality rates as those reported in ref. 17 (see Supplementary Fig. S1). For the other case studies, *t* was 4.2 years for Tapajós, 7 years for Caxiuanã, and 22 weeks (0.42 years) in the seedling experiment in Panama.

In the experiment in Bolivia, water supply was suppressed for 40 individuals of each species^12^. The only information available was *t*, the time to death for each species, i.e. the number of days after irrigation stopped when all 40 individuals of each species had died. Therefore, at time *t*, the number of individuals, *n*_*t*_, would be 0, and mortality (*m*) would necessarily be equal to 1 for all species. In order to calculate mortality rate using the data from ref. 12, we assumed that on the day before all individuals were reported as dead, only one individual remained. Thus, we applied Eq. 2 at time *t* − 1, where *n*_*t*_ = 1 so that different values of mortality rate per species could be assessed. We scaled the information to the genus level, considering *n*_0_ as the sum of all individuals within congeneric species and *t* as the maximum *t* among the species within each genus.

Selecting the appropriate taxonomic unit for analysis is necessarily a compromise between maximising replication within units and the need to have sufficient degrees of freedom in terms of the number of units. After preliminary exploration of such effects (see Supplementary methods S2), we elected to work at the genus level. This helps maximize the characterization of the tree community. For example, among all taxa in Tapajós, 98% of tree genera had information on WDA, while only 57% of tree species appear in both the mortality experiment and the WDA datasets (see Supplementary methods S2 and Supplementary Table S3). Furthermore, the average number of individuals for each genus is naturally larger than for species, providing more confidence when calculating mortality. Taxonomic names from all data sets were standardized against the Tropicos database using the Taxonomic Name Resolution Service^68^. All analyses were carried out in R version 3.1.2^69^.

### Controlling for the baseline mortality

Within any forest, stand mortality rates vary, with population dynamics differing from genus to genus e.g. refs 67, 70. Therefore, before testing the influence of a disturbance or any stressor on mortality rates, it is important to determine the baseline mortality of each genus (i.e. its mortality under standard conditions, which in the through-fall exclusion experiments refers to mortality in the control areas and in BCI to the post-drought interval). Here we estimated a ‘drought-induced mortality’ ($\bigtriangleup {\rm{m}}$), or drought anomaly, by simply subtracting the baseline mortality rate from the mortality rate under drought conditions (Eq. 3).

3 $${\rm{\Delta }}m={m}_{drought}-{m}_{non-drought}$$

The seedling experiment in Bolivia lacked a control treatment, therefore it is not possible to control for baseline mortality, and in this case, analyses were performed using the mortality calculated as described in Eq. 2.

### Statistical approach

For each experiment, we assessed the relationship between drought-induced mortality and climate affiliation. For Caxiuanã, BCI and Tapajós, where the number of stems per genus was in some cases potentially too small to accurate estimate of mortality rates, we first investigate the influence of these rare taxa on the correlation between ∆m and WDA (see Supplementary methods S2). This investigation showed the inherent trade-off between the number of stems per taxa that allows to accurate estimate mortality rates and the number of taxa necessary to detect the relationship between ∆m and WDA. Given this we selected the subset that optimises our ability to detect the relationship between ∆m and WDA (see Supplementary methods S2). For the selected subset (115 out of the 208 genera across all experiments), we tested the relationship between ∆m and WDA using Kendall’s τ coefficient of correlation and standardized major axis regression (SMA)^71^ using the R package *smatr* ^72^. One-tail P-values were calculated to explicitly test the null hypotheses of non-positive correlation between ∆m and WDA.

Next, we combined studies, where appropriate, to test whether an overarching WDA effect was evident. This was done in two ways. Firstly, we analysed all four different datasets with controls together (BCI, Tapajós, Caxiuanã and Panama seedling experiment), by standardizing ∆m into z-scores within each experiment and using Bayesian phylogenetic mixed models to explain ∆m. WDA, experiment and their interaction were included as fixed effects. Secondly, for the two largest data sets only, BCI and Tapajós, which included information on trees and saplings, we used Bayesian phylogenetic mixed models as before to understand the influence of WDA on ∆m, also here investigating the influence of life stage on this relationship. We included experiment, WDA and life stages as fixed effects. For this analysis, values of ∆m were transformed to log (∆m + 0.1) to minimise heteroscedasticity in the residuals. In both analytical frameworks, use of the Bayesian phylogenetic mixed models allowed us to account for phylogenetic autocorrelation as a random effect and thus take into account the non-independence among genera as a consequence of their shared phylogenetic history^73, 74^. Our models were developed using the R package *MCMCglmm* ^75^. To select among models we used the deviance information criteria (DIC), a Bayesian equivalent of Akaike’s Information Criteria^76^. The phylogenetic information was obtained from a phylogeny developed for the genus level by ref. 77, which includes 632 Amazonian tree genera. The model requires each data point to be a separate branch in the phylogeny, thus for the cases where genera were repeated across studies they were considered as polytomies.

## Electronic supplementary material

Supplementary information
