# Supplementary material for: Biogeographic distributions of neotropical trees reflect their directly measured drought tolerances
Source: Sci Rep. 2017 Aug 21;7:8334. doi: 10.1038/s41598-017-08105-8 (PMC5567183; doi:10.1038/s41598-017-08105-8)
Supplement: Supplementary file 1 — Supplementary information [file 41598_2017_8105_MOESM1_ESM.pdf]

Biogeographic distributions of neotropical trees reflect their directly measured drought tolerances

Adriane Esquivel-Muelbert[1]*, David Galbraith[1], Kyle G. Dexter[2,3], Timothy R. Baker[1], Simon L. Lewis[1,4], Patrick Meir[3,5], Lucy Rowland[3,6], Antonio Carlos Lola da Costa[7], Daniel Nepstad[8] & Oliver L. Phillips[1]

1. School of Geography, University of Leeds, Leeds, LS2 9JT, UK

2. Royal Botanic Garden of Edinburgh, EH3 5LR, Edinburgh, UK

3. School of Geosciences, University of Edinburgh, Edinburgh, UK

4. Department of Geography, University College London, London, UK

5. Research School of Biology, Australian National University, Canberra, Australia.

6. Department of Geography, College of Life and Environmental Sciences, University of Exeter, Exeter, UK

7. Universidade Federal do Pará, Belém, Pará, Brazil

8. Earth Innovation Institute, San Francisco, CA, USA.

* adriane.esquivel@gmail.com

Supplementary Fig. S1. Correlation between mortality rates in [1] and as calculated in this study. The x-axes show mortality rates (MR) as calculated by [1] and the y-axes represent mortality rates as calculated in this study. The left graph shows mortality rates at the drought period (1982-85) and the right graph mortality rates in the control period (1985 – 1990).

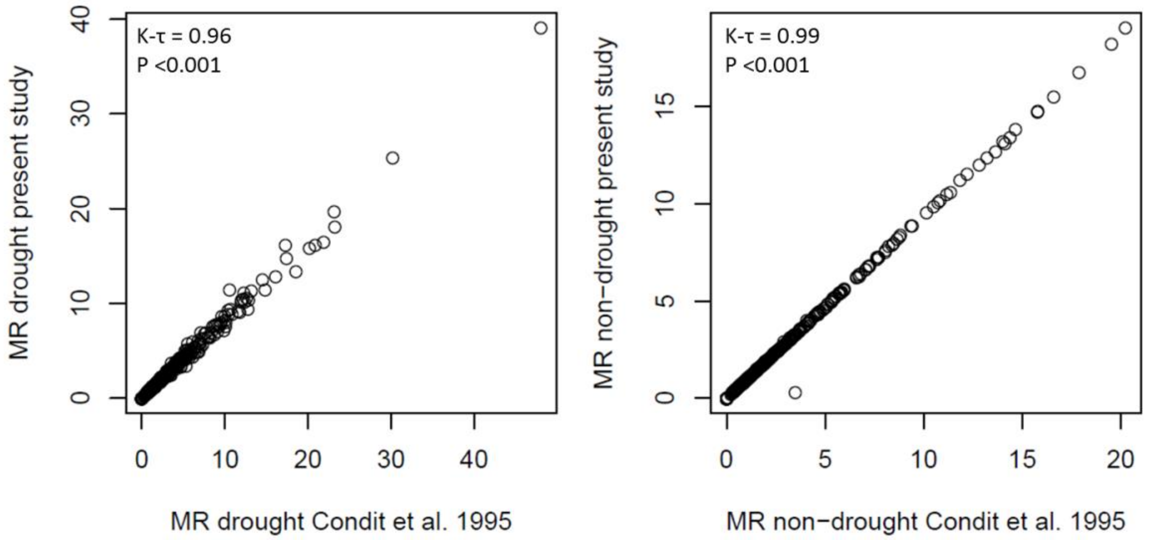

Supplementary methods S2. Accounting for the effect of sample size on the correlation between drought-induced mortality (Δm) and water deficit affiliation (WDA).

To assess the impact of (small) sample sizes of individual taxa on confounding our mortality rates estimates (Δm) vs. WDA values, we performed a series of exploratory analyses using all available data from BCI, Caxiuanã and Tapajós. We calculated these correlations for different subsamples, varying the minimum number of stems per taxon required for the taxon to be included in each subsample (Figures S2.1 to S2.5). This gives information on how the number of stems observed per taxon and the number of taxa analysed in each study case can, together, affect the correlation statistics between drought tolerance and WDA for Tapajós, Caxiuanã and BCI.

When analysing the influence of the number of stems in the correlation, values of Kendall τ tend to clearly increase as the analyses become progressively restricted to only the best sampled taxa (Figures S2.1 to S2.5). However, as the sample size in terms of number of taxa inevitably declines when these criteria are heightened, the P-values for evaluating the hypothesis of association between Δm and WDA tend to be minimal at intermediate numbers of taxa. This happens as a consequence of the statistical power available to test the hypothesis being maximal when the number of taxa with an adequate number of individuals to estimate mortality accurately is sufficient to test for the relationship between Δm and WDA. We use the subset that maximizes the correlation between Δm and WDA. Note that the direction of the relationship between Δm and WDA is consistently positive across the different subsets regardless of the sample size.

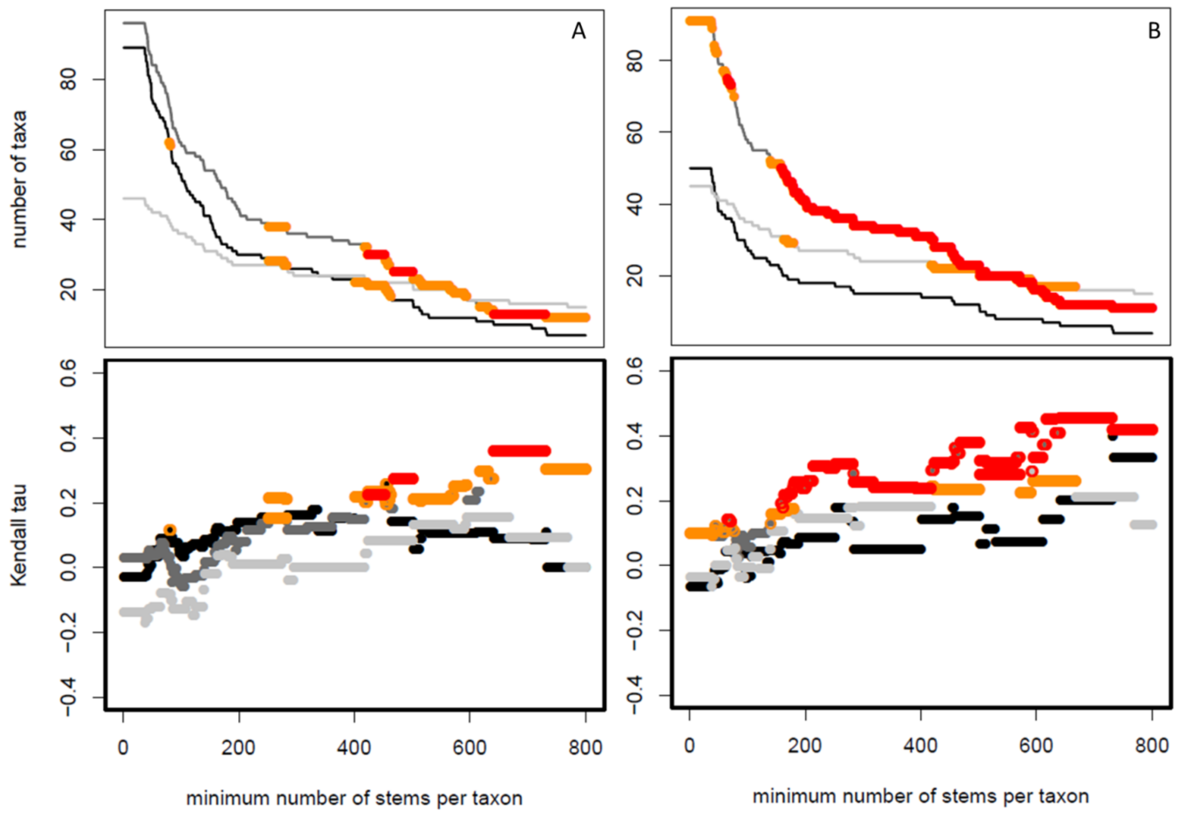

Figure S2.1 – Influence of sample sizes on correlation between drought-induced mortality (Δm) and water deficit affiliation (WDA) of trees > 100 mm D as a consequence of the 1982-83 drought in BCI (Condit et al., 1995). Here we present values of Kendall's τ coefficient of correlation between Δm and WDA and the P value of each correlation for various subsamples from the original data, differing in the minimum number of stems per taxa. The subsamples are ordered by the minimum number of stems per taxa, moving from 1 to 800, along each x-axis. The top graphs show the number of taxa within each subsample. The bottom graphs show values of Kendall's τ coefficient of correlation between Δm and WDA for each subsample. Black dots represent analyses performed at the species, dark grey at the genus and light grey at the family level. The two graphs on the left show analyses including all taxa whilst the graphs on the right show results for excluding taxa affiliated to locally enhanced water supply areas. P values test the null hypothesis of no positive relationship between Δm and WDA, considering $\alpha = 0.1$ (orange dots) and $\alpha = 0.05$ (red dots).

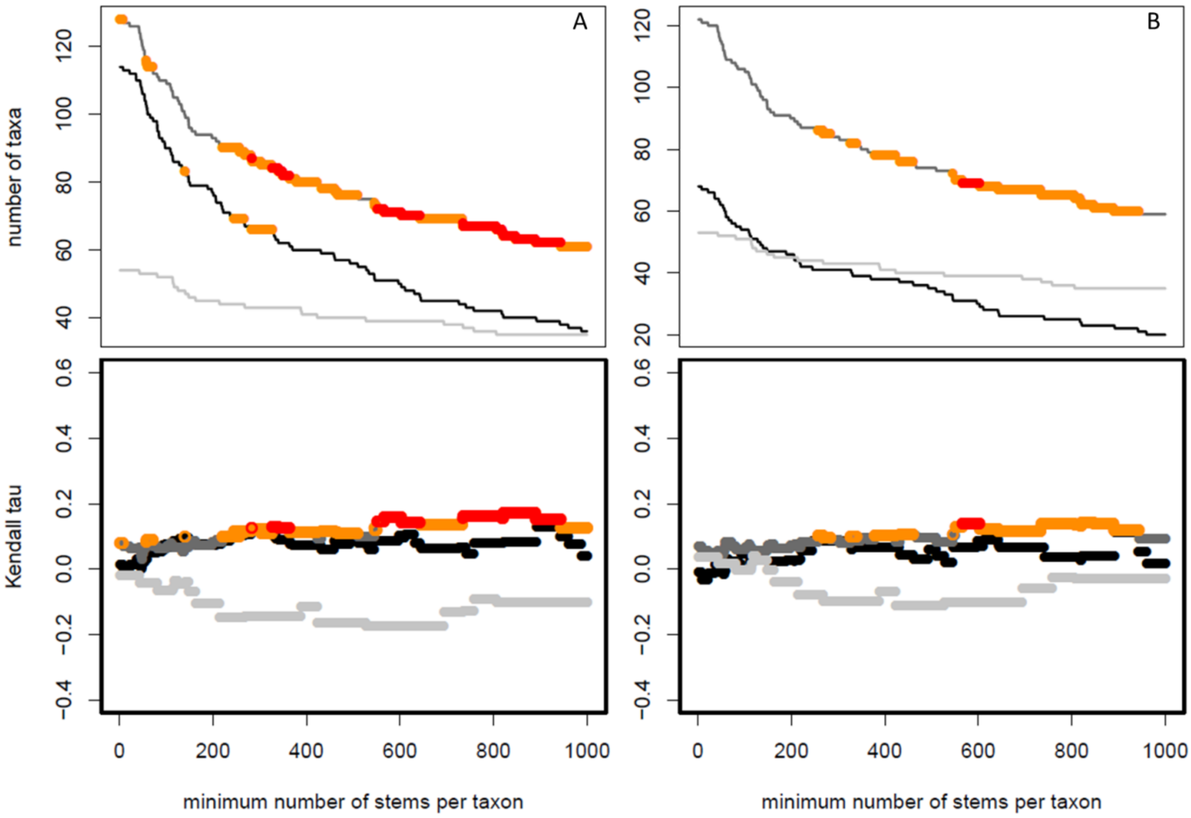

Figure S2.2 – Influence of sample sizes on the correlation between drought-induced mortality (Δm) and water deficit affiliation (WDA) of saplings 10 mm – 99 mm D as a consequence of the 1982-83 drought in BCI (Condit et al., 1995). Here we present values of Kendall's τ coefficient of correlation between Δm and WDA and the P value of each correlation for various subsamples from the original data, differing in the minimum number of stems per taxa. The subsamples are ordered by the minimum number of stems per taxa, moving from 1 to 1000 along each x-axis. The top graphs show the number of taxa within each subsample. The bottom graphs show values of Kendall's τ coefficient of correlation between Δm and WDA for each subsample. Black dots represent analyses performed at the species, dark grey at the genus and light grey at the family level. The two graphs on the left show analyses including all taxa whilst the graphs on the right show results for excluding taxa affiliated to locally enhanced water supply areas. P values test the null hypothesis of no positive relationship between Δm and WDA, considering $\alpha = 0.1$ (orange dots) and $\alpha = 0.05$ (red dots).

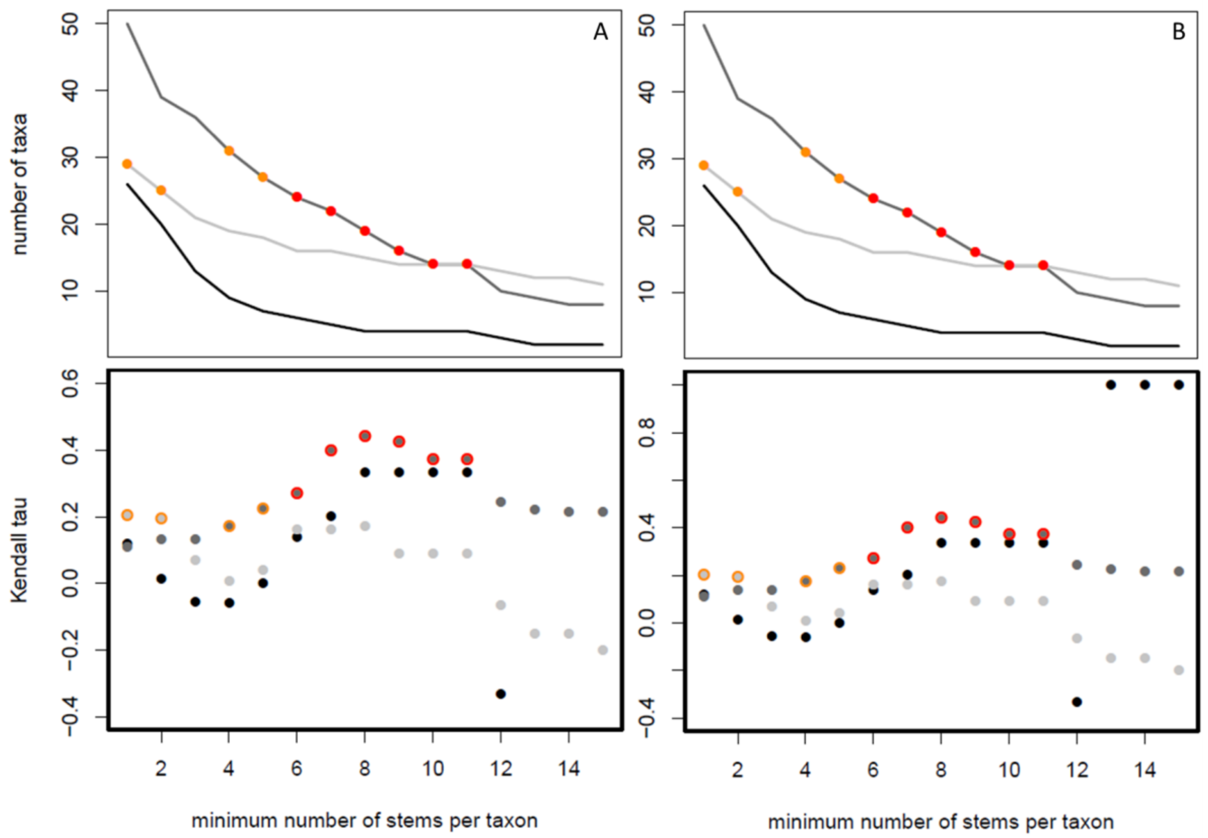

Figure S2.3 Influence of sample sizes on correlation between drought-induced mortality effect (Δm) and precipitation affiliation for trees > 100 mm D in the Tapajós drought experiment [2]. Here we present values of Kendall's τ coefficient of correlation between Δm and WDA and the P value of each correlation for various subsamples from the original data differing in the minimum number of stems per taxa. The subsamples are ordered by the minimum number of stems per taxa, moving from 1 to 14 along each x-axis. The top graphs show the number of taxa within each subsample. The bottom graphs show values of Kendall's τ coefficient of correlation between Δm and WDA for each subsample. Black dots represent analyses performed at the species, dark grey at the genus and light grey at the family level. The two graphs on the left show analyses including all taxa whilst the graphs on the right show results for excluding taxa affiliated to locally enhanced water supply areas. P values test the null hypothesis of no positive relationship between Δm and WDA, considering $\alpha = 0.1$ (orange dots) and $\alpha = 0.05$ (red dots).

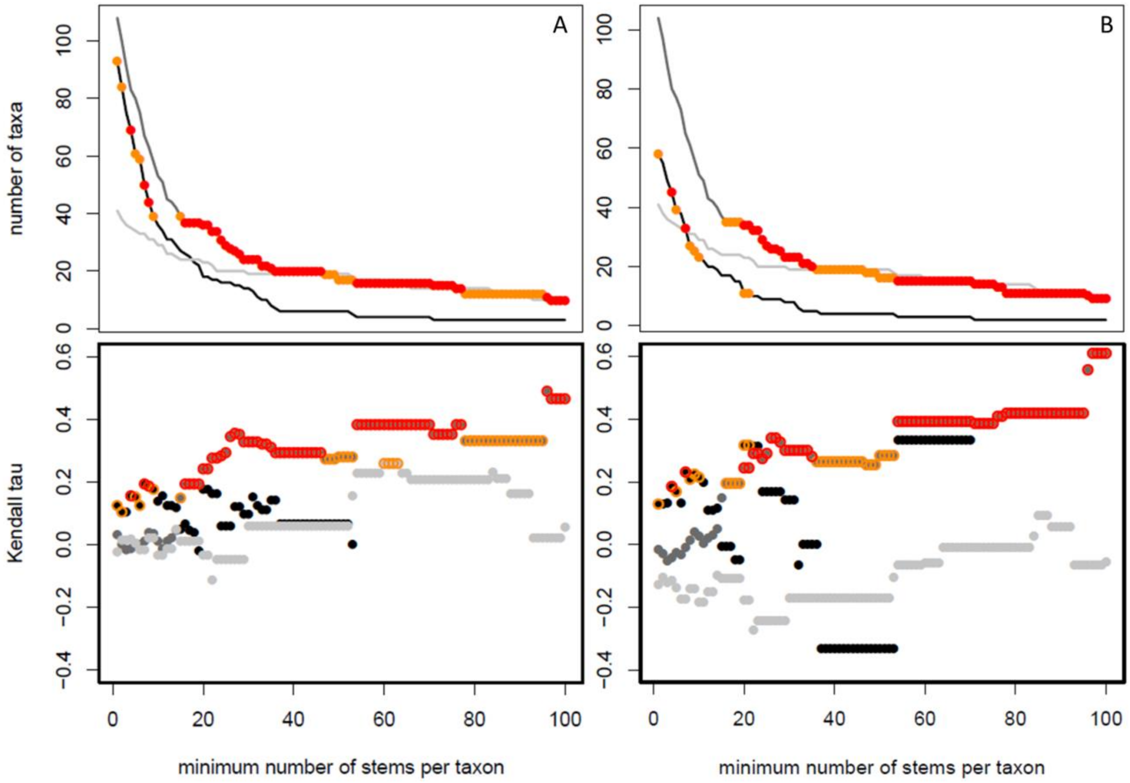

Figure S2.4 Influence of sample sizes on correlation between drought-induced mortality effect (Δm) and precipitation affiliation for saplings (20 – 99 mm D) in Tapajós drought experiment [2]. Here we present values of Kendall's τ coefficient of correlation between Δm and WDA and the P value of each correlation for various subsamples from the original data differing in the minimum number of stems per taxa. The subsamples are ordered by the minimum number of stems per taxa, moving from 1 to 100 along each x-axis. The top graphs show the number of taxa within each subsample. The bottom graphs show values of Kendall's τ coefficient of correlation between Δm and WDA for each subsample. Black dots represent analyses performed at the species, dark grey at the genus and light grey at the family level. The two graphs on the left show analyses including all taxa whilst the graphs on the right show results for excluding taxa affiliated to locally enhanced water supply areas. P values test the null hypothesis of no positive relationship between Δm and WDA, considering $\alpha = 0.1$ (orange dots) and $\alpha = 0.05$ (red dots).

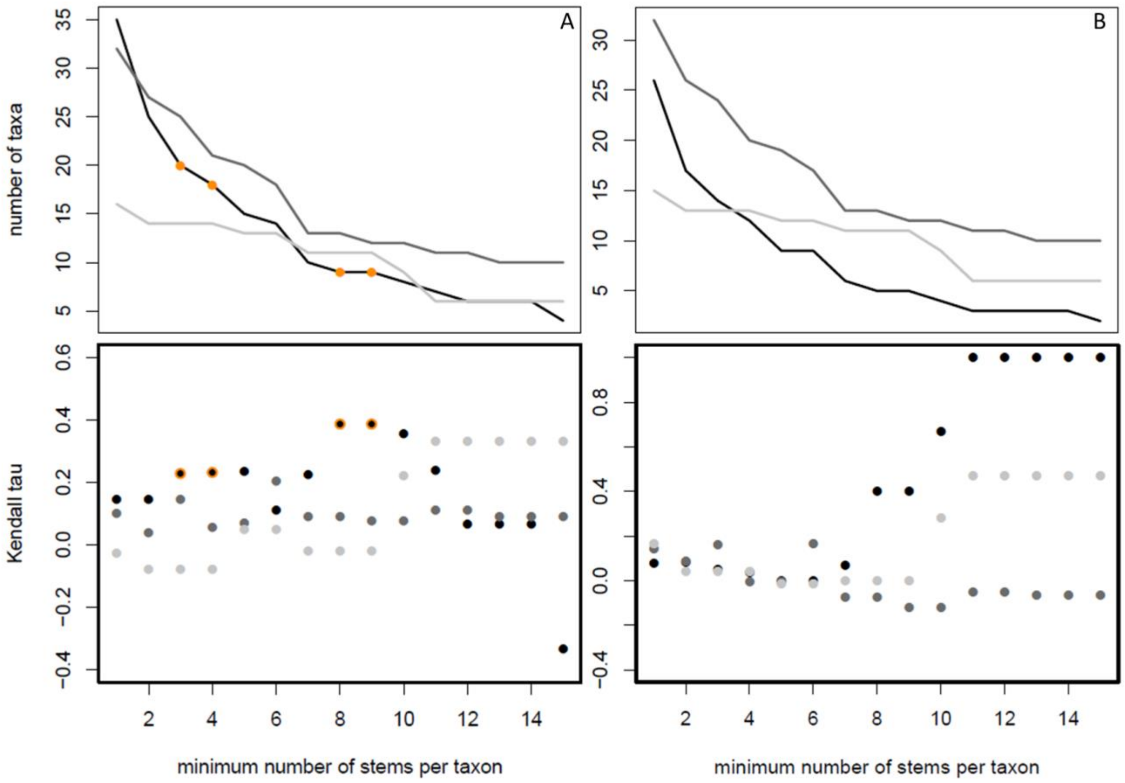

Figure S2.5 - Influence of sample sizes on the correlation between drought-induced mortality effect (Δm) and precipitation affiliation for trees (>10cm D) in the Caxiuanã drought experiment [3]. Here we present values of Kendall's τ coefficient of correlation between Δm and WDA and the P value of each correlation for various subsamples from the original data differing in the minimum number of stems per taxa. The subsamples are ordered by the minimum number of stems per taxa, moving from 1 to 1000 along each x-axis. The top graphs show the number of taxa within each subsample. The bottom graphs show values of Kendall's τ coefficient of correlation between Δm and WDA for each subsample. Black dots represent analyses performed at the species, dark grey at the genus and light grey at the family level. The two graphs on the left show analyses including all taxa whilst the graphs on the right show results for excluding taxa affiliated to locally enhanced water supply areas. P values test the null hypothesis of no positive relationship between Δm and WDA, considering α = 0.1 (orange dots) and α = 0.05 (red dots).

Supplementary Table S3 Number and proportions of taxa from original studies analysed. Number of taxa in the original study represents the number of taxa occurring in both drought and control observations. The number of taxa from the original case studies that had information on WDA are shown also as a percentage of the number of taxa in the original study in brackets. Values are shown for all taxa and for a subset excluding taxa affiliated with areas of locally enhanced water supply (LEWS).

Source	Taxonomic level	Number of taxa in original study	Number taxa with information on WDA	
			All taxa (%)	Excluding LEWS affiliated (%)
BCI trees	Species	132	89 (67)	50 (38)
	Genera	99	96 (97)	91 (92)
	Families	46	46 (100)	45 (98)
Tapajós trees	Species	70	40 (57)	26 (37)
	Genera	53	52 (98)	50 (94)
	Families	29	29(100)	29 (100)
Caxiuanã	Species	63	35 (55)	26 (41)
	Genera	36	32 (89)	32 (89)
	Families	16	16 (100)	15 (94)
BCI saplings	Species	203	116 (57)	68 (33)
	Genera	143	130 (90)	124 (87)
	Families	54	54 (100)	53 (98)
Tapajós saplings	Species	202	100 (49)	64 (32)
	Genera	119	114 (96)	110 (92)
	Families	43	43 (100)	43 (100)
Poorter & Markesteijn (2008)	Species	36	30 (83)	18 (50)
	Genera	31	29 (93)	26 (84)
	Families	18	18(100)	17(94)
Engelbrecht et al. (2007)	Species	48	29 (60)	16 (33)
	Genera	45	41 (91)	40 (88)
	Families	29	29(100)	29(100)

Supplementary Table S4 Mortality rates at drought and control observations for trees and saplings in the different case studies calculated across individuals and at the genus-levels. Means were calculated considering the genera that occur in both, control and droughted areas. Values in brackets represent standard deviations. Mortality rates are calculated for BCI, the 1982-3 El Niño drought in Barro Colorado Island in Panama [1] and two through-fall exclusion experiments in the Brazilian Amazon, Tapajós [2] (TAP) and Caxiuanã [3] (CAX).

		$m_{control}$	$m_{\mathrm{droughted}}$	$m_{\mathrm{droughted}}$ / $m_{control}$	$\Delta(m_{\mathrm{droughted}}$ - $m_{control})$
mean *m* all individuals					
Trees	CAX	0.8	2.4	2.9	1.6
	TAP	1.9	5.3	2.8	3.4
	BCI	2.0	2.3	1.2	0.3
Saplings	TAP	1.9	2.2	1.8	0.3
	BCI	2.2	2.2	1	-0.1
mean *m* per genus					
Trees	all observations	4.8 (8)	7 (20)	3.2	4.8
	CAX	0.8 (2)	10.1 (27)	13.4	9.4 (27)
	TAP	2.7 (14)	13.1 (29)	4.8	10.4 (33)
	BCI	2.4 (3)	2.6 (2)	1	0.1 (1)
Saplings					
	TAP	3.7 (16)	4.5 (16)	1.2	0.8 (19)
	BCI	3.4 (4)	3.6 (4)	1.1	0.2 (2)

Supplementary Table S5 Drought-induced mortality and water deficit affiliation (mm) data for all genera in different experiments and at different life history stages.

Genus	Drought-induced mortality	Water deficit affiliation (mm)	Experiment	Life-history stage
Alibertia	0.40	-397	Panama	seedlings
Anaxagorea	0.29	-39	Panama	seedlings
Andira	0.16	-134	Panama	seedlings
Beilschmiedia	0.22	-204	Panama	seedlings
Brosimum	0.62	-183	Panama	seedlings
Calophyllum	0.57	-146	Panama	seedlings
Capparidastrum	0.00	-122	Panama	seedlings
Casearia	0.08	-177	Panama	seedlings
Cordia	0.29	-245	Panama	seedlings
Cupania	0.26	-297	Panama	seedlings
Dipteryx	0.00	-227	Panama	seedlings
Faramea	0.00	-258	Panama	seedlings
Garcinia	0.00	-139	Panama	seedlings
Guapira	0.00	-322	Panama	seedlings
Guarea	0.93	-123	Panama	seedlings
Hymenaea	0.23	-89	Panama	seedlings
Inga	0.26	-105	Panama	seedlings
Lacistema	0.78	-141	Panama	seedlings
Lacmellea	0.29	-49	Panama	seedlings
Licania	0.48	-52	Panama	seedlings
Mouriri	0.07	-62	Panama	seedlings
Myrcia	0.63	-147	Panama	seedlings
Ouratea	0.08	-152	Panama	seedlings
Picramnia	0.47	-105	Panama	seedlings
Piper	0.83	-197	Panama	seedlings
Posoqueria	0.08	-151	Panama	seedlings
Pouteria	0.45	-158	Panama	seedlings
Pseudobombax	0.19	-314	Panama	seedlings
Psychotria	0.07	-148	Panama	seedlings
Pterocarpus	0.56	-121	Panama	seedlings
Sorocea	0.78	-103	Panama	seedlings
Swartzia	0.00	-86	Panama	seedlings
Tabebuia	0.71	-337	Panama	seedlings
Tetragastris	0.34	-138	Panama	seedlings
Trichilia	0.48	-196	Panama	seedlings
Unonopsis	0.89	-38	Panama	seedlings
Virola	0.77	-67	Panama	seedlings
Vochysia	0.65	-197	Panama	seedlings
Xylopia	0.91	-245	Panama	seedlings
Xylosma	0.41	-205	Panama	seedlings

Cont. Table S5

Genus	Drought-induced mortality	Water deficit affiliation (mm)	Experiment	Life-history stage
Alseis	0.00	-184	BCI	trees
Cordia	0.00	-245	BCI	trees
Faramea	0.00	-258	BCI	trees
Guarea	0.00	-123	BCI	trees
Gustavia	0.00	-31	BCI	trees
Hirtella	0.00	-190	BCI	trees
Oenocarpus	0.00	-108	BCI	trees
Protium	0.01	-151	BCI	trees
Quararibea	0.01	-146	BCI	trees
Socratea	0.00	-201	BCI	trees
Trichilia	0.00	-196	BCI	trees
Virola	0.01	-67	BCI	trees
Acalypha	0.00	-110	BCI	saplings
Alibertia	0.00	-397	BCI	saplings
Alseis	0.00	-184	BCI	saplings
Anaxagorea	0.02	-39	BCI	saplings
Annona	0.01	-235	BCI	saplings
Aspidosperma	0.00	-234	BCI	saplings
Beilschmiedia	0.00	-204	BCI	saplings
Brosimum	0.00	-183	BCI	saplings
Calophyllum	0.00	-146	BCI	saplings
Capparidastrum	0.00	-122	BCI	saplings
Casearia	0.01	-177	BCI	saplings
Cassipourea	0.00	-169	BCI	saplings
Chrysochlamys	0.00	-38	BCI	saplings
Chrysophyllum	0.00	-131	BCI	saplings
Coccoloba	0.00	-213	BCI	saplings
Cordia	0.00	-245	BCI	saplings
Coussarea	0.00	-90	BCI	saplings
Croton	-0.02	-179	BCI	saplings
Cupania	0.00	-297	BCI	saplings
Drypetes	0.00	-179	BCI	saplings
Erythroxylum	0.00	-221	BCI	saplings
Eugenia	0.00	-122	BCI	saplings
Faramea	0.00	-258	BCI	saplings
Garcinia	-0.01	-139	BCI	saplings
Guarea	0.00	-123	BCI	saplings
Guatteria	0.00	-76	BCI	saplings
Guettarda	-0.01	-290	BCI	saplings
Heisteria	0.00	-204	BCI	saplings
Hirtella	0.00	-190	BCI	saplings
Inga	0.00	-105	BCI	saplings

Cont. Table S5

Genus	Drought-induced mortality	Water deficit affiliation (mm)	Experiment	Life-history stage
Lacistema	0.00	-141	BCI	saplings
Laetia	-0.01	-222	BCI	saplings
Licania	0.00	-52	BCI	saplings
Lonchocarpus	0.00	-276	BCI	saplings
Maquira	0.00	-83	BCI	saplings
Miconia	-0.02	-155	BCI	saplings
Mouriri	-0.01	-62	BCI	saplings
Nectandra	0.02	-193	BCI	saplings
Ocotea	0.01	-270	BCI	saplings
Oenocarpus	0.01	-108	BCI	saplings
Ouratea	0.00	-152	BCI	saplings
Palicourea	-0.03	-251	BCI	saplings
Pentagonia	0.01	-3	BCI	saplings
Picramnia	0.00	-105	BCI	saplings
Piper	-0.03	-197	BCI	saplings
Pouteria	0.00	-158	BCI	saplings
Protium	0.00	-151	BCI	saplings
Psychotria	-0.01	-148	BCI	saplings
Pterocarpus	0.00	-121	BCI	saplings
Quararibea	0.00	-146	BCI	saplings
Randia	0.00	-268	BCI	saplings
Rinorea	0.00	-32	BCI	saplings
Simarouba	-0.01	-206	BCI	saplings
Siparuna	0.02	-170	BCI	saplings
Sloanea	0.00	-171	BCI	saplings
Socratea	-0.01	-201	BCI	saplings
Sorocea	0.00	-103	BCI	saplings
Stylogyne	0.00	-147	BCI	saplings
Swartzia	0.00	-86	BCI	saplings
Tabernaemontana	0.00	-262	BCI	saplings
Tachigali	-0.01	-193	BCI	saplings
Talisia	0.00	-257	BCI	saplings
Tetragastris	0.00	-138	BCI	saplings
Trichilia	0.00	-196	BCI	saplings
Trophis	0.00	-252	BCI	saplings
Unonopsis	0.00	-38	BCI	saplings
Virola	0.00	-67	BCI	saplings
Xylopia	0.00	-245	BCI	saplings
Zanthoxylum	-0.02	-271	BCI	saplings
Aparisthmium	0.98	-35	Tapajós	trees
Brosimum	0.00	-183	Tapajós	trees
Cordia	-0.04	-245	Tapajós	trees
Couratari	-0.04	-208	Tapajós	trees

Cont. Table S5

Genus	Drought-induced mortality	Water deficit affiliation (mm)	Experiment	Life-history stage
Coussarea	0.01	-90	Tapajós	trees
Erisma	0.05	-104	Tapajós	trees
Eschweilera	0.02	-47	Tapajós	trees
Inga	0.19	-105	Tapajós	trees
Iryanthera	0.15	-101	Tapajós	trees
Lecythis	0.00	-55	Tapajós	trees
Licania	1.00	-52	Tapajós	trees
Licaria	0.13	-140	Tapajós	trees
Miconia	-0.01	-155	Tapajós	trees
Myrciaria	0.00	-320	Tapajós	trees
Pouteria	0.02	-158	Tapajós	trees
Protium	-0.01	-151	Tapajós	trees
Tachigali	0.04	-193	Tapajós	trees
Virola	0.00	-67	Tapajós	trees
Zygia	0.28	-25	Tapajós	trees
Amphiodon	0.00	-80	Tapajós	saplings
Aparisthmium	0.01	-35	Tapajós	saplings
Coussarea	0.01	-90	Tapajós	saplings
Faramea	-0.03	-258	Tapajós	saplings
Inga	0.03	-105	Tapajós	saplings
Protium	0.01	-151	Tapajós	saplings
Rinorea	0.03	-32	Tapajós	saplings
Tachigali	0.00	-193	Tapajós	saplings
Talisia	0.00	-257	Tapajós	saplings
Acacia	0.16	-284	Bolivia	seedlings
Alibertia	0.14	-397	Bolivia	seedlings
Amburana	0.02	-469	Bolivia	seedlings
Anadenanthera	0.15	-575	Bolivia	seedlings
Aspidosperma	0.16	-234	Bolivia	seedlings
Batocarpus	0.18	-58	Bolivia	seedlings
Cariniana	0.16	-258	Bolivia	seedlings
Cedrela	0.15	-354	Bolivia	seedlings
Ceiba	0.03	-383	Bolivia	seedlings
Geissanthus	0.18	-602	Bolivia	seedlings
Hymenaea	0.28	-89	Bolivia	seedlings
Licaria	0.25	-140	Bolivia	seedlings
Margaritaria	0.20	-189	Bolivia	seedlings
Myracrodruon	0.17	-586	Bolivia	seedlings
Myrciaria	0.18	-320	Bolivia	seedlings
Platymiscium	0.14	-222	Bolivia	seedlings
Pseudobombax	0.03	-314	Bolivia	seedlings
Pseudolmedia	0.21	-216	Bolivia	seedlings
Sapindus	0.16	-507	Bolivia	seedlings

Cont. Table S5

Genus	Drought-induced mortality	Water deficit affiliation (mm)	Experiment	Life-history stage
Sapium	0.18	-297	Bolivia	seedlings
Spondias	0.03	-264	Bolivia	seedlings
Sweetia	0.16	-616	Bolivia	seedlings
Swietenia	0.25	-282	Bolivia	seedlings
Tabebuia	0.16	-337	Bolivia	seedlings
Trema	0.25	-242	Bolivia	seedlings
Triplaris	0.37	-187	Bolivia	seedlings
Aspidosperma	0.00	-234	Caxiuanã	trees
Chrysophyllum	0.03	-131	Caxiuanã	trees
Duguetia	-0.04	-191	Caxiuanã	trees
Eschweilera	0.03	-47	Caxiuanã	trees
Inga	0.03	-105	Caxiuanã	trees
Lecythis	0.01	-55	Caxiuanã	trees
Licania	0.01	-52	Caxiuanã	trees
Manilkara	0.02	-255	Caxiuanã	trees
Micropholis	0.05	-93	Caxiuanã	trees
Minquartia	-0.04	-31	Caxiuanã	trees
Ouratea	0.04	-152	Caxiuanã	trees
Pouteria	0.01	-158	Caxiuanã	trees
Protium	0.02	-151	Caxiuanã	trees
Stachyarrhena	0.02	-4	Caxiuanã	trees
Swartzia	0.01	-86	Caxiuanã	trees
Tetragastris	-0.03	-138	Caxiuanã	trees
Zygia	0.04	-25	Caxiuanã	trees

Supplementary Table S6 One and two-tailed P-values for the relationship between drought mortality (Δm) and water deficit affiliation (WDA) in five droughted locations in the Neotropics. Kendall's τ coefficient of correlation (Kτ) between Δm and WDA were calculated for the 1982-3 El Niño drought in BCI [1], two through-fall exclusion experiments in the Brazilian Amazon, Tapajós [2] and Caxiuanã [3], and two seedling experiments, one in Panama [4] the other in Bolivia [5]. For BCI, Tapajós and Caxiuanã the relationship Δm *vs.* WDA was calculated for the subset (Number of genera) that maximizes detectability, including only taxa with the minimum number of stems (Min. number of stem per taxa) needed to permit estimation of mortality for that experiment (see Supplementary methods S2 for details). The minimum number of stems per taxa varied depending on the duration of the drought.

Source	Life stage	Kτ	1-sided P-value	2-sided P-value	Number of genera	Min. number of stems per taxa
BCI	trees	0.42	0.02	0.04	12	730
	saplings	0.13	0.04	0.09	74	602
Tapajós	trees	0.44	0.004	0.009	19	8
	saplings	0.61	0.01	0.02	9	100
Caxiuanã	trees	0.16	0.18	0.36	6	17
Panama	seedlings	0.05	0.32	0.65	40	53
Bolivia	seedlings	0.32	0.01	0.02	31	40

Note that the overall results are not affected when using 2-sided P-values. The only case where results change from being significant to marginally significant was for BCI saplings.

Supplementary Fig. S7

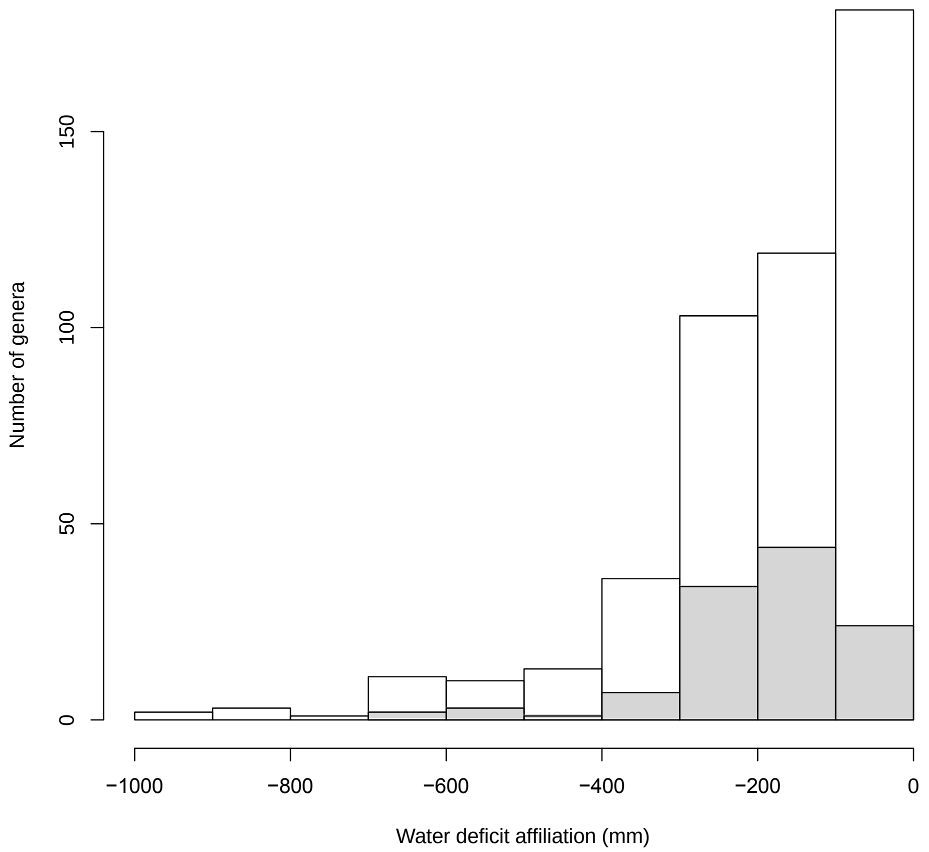

Fig. S7 - Histogram comparing water deficit affiliation of 115 genera in our analyses (grey) with most Amazonian genera (white)[6]. Note that while most genera are affiliated to aseasonal conditions (WDA between 0 and -100 mm y^{-1}) the majority of genera in drought-experiments are affiliated to seasonal climate (WDA between -100 and -300 mm y^{-1}).

References

1 Condit, R., Hubbell, S. P. & Foster, R. B. Mortality-rates of 205 neotropical tree and shrub species and the impact of a severe drought. *Ecological Monographs* **65**, 419-439, doi:10.2307/2963497 (1995).

2 Nepstad, D. C., Tohver, I. M., Ray, D., Moutinho, P. & Cardinot, G. Mortality of large trees and lianas following experimental drought in an amazon forest. *Ecology* **88**, 2259-2269, doi:10.1890/06-1046.1 (2007).

3 da Costa, A. C. L. *et al.* Effect of 7 yr of experimental drought on vegetation dynamics and biomass storage of an eastern Amazonian rainforest. *New Phytol.* **187**, 579-591, doi:10.1111/j.1469-8137.2010.03309.x (2010).

4 Engelbrecht, B. M. J. *et al.* Drought sensitivity shapes species distribution patterns in tropical forests. *Nature* **447**, 80-U82, doi:10.1038/nature05747 (2007).

5 Poorter, L. & Markesteijn, L. Seedling traits determine drought tolerance of tropical tree species. *Biotropica* **40**, 321-331, doi:10.1111/j.1744-7429.2007.00380.x (2008).

6 Esquivel-Muelbert, A. *et al.* Seasonal drought limits tree species across the Neotropics. *Ecography* **40**, 618-629, doi:10.1111/ecog.01904 (2017).
